# Elevated ganglioside GM2 activator (GM2A) in human brain tissue reduces neurite integrity and spontaneous neuronal activity

**DOI:** 10.1186/s13024-022-00558-4

**Published:** 2022-09-21

**Authors:** Yi-Chen Hsieh, Joseph Negri, Amy He, Richard V. Pearse, Lei Liu, Duc M. Duong, Lori B. Chibnik, David A. Bennett, Nicholas T. Seyfried, Tracy L. Young-Pearse

**Affiliations:** 1 Ann Romney Center for Neurologic Diseases, Brigham and Women’s Hospital and Harvard Medical School, 60 Fenwood Rd, Boston, MA 02115 USA; 2 Department of Biochemistry, Emory University School of Medicine, 1510 Clifton Rd NE, Atlanta, GA 30322 USA; 3 Department of Neurology, Massachusetts General Hospital, 55 Fruit St, Boston, MA 02114 USA; 4 Department of Epidemiology, Harvard T.H. Chan School of Public Health, 677 Huntington Ave, Boston, MA 02115 USA; 5 Rush Alzheimer’s Disease Center, Rush University Medical Center, 600 S. Paulina St, Chicago, IL 60612 USA; 6 Department of Neurology, Emory University School of Medicine, 100 Woodruff Circle, Atlanta, GA 30322 USA; 7 Harvard Stem Cell Institute, Harvard University, 7 Divinity Ave, Cambridge, MA 02138 USA

**Keywords:** Alzheimer’s, LOAD, Aβ, Tau, GM2A, WAVE, ABI1, ABI2, HEXA, MEA, Proteomics, Sex

## Abstract

**Background:**

Alzheimer’s Disease (AD) affects millions globally, but therapy development is lagging. New experimental systems that monitor neuronal functions in conditions approximating the AD brain may be beneficial for identifying new therapeutic strategies.

**Methods:**

We expose cultured neurons to aqueous-soluble human brain extract from 43 individuals across a spectrum of AD pathology. Multi-electrode arrays (MEAs) and live-cell imaging were used to assess neuronal firing and neurite integrity (NI), respectively, following treatments of rat cortical neurons (MEA) and human iPSC-derived neurons (iN) with human brain extracts.

**Results:**

We observe associations between spontaneous activity and Aβ42:40 levels, between neurite integrity and oligomeric Aβ, and between neurite integrity and tau levels present in the brain extracts. However, these associations with Aβ and tau do not fully account for the effects observed. Proteomic profiling of the brain extracts revealed additional candidates correlated with neuronal structure and activity. Neurotoxicity in MEA and NI assays was associated with proteins implicated in lysosomal storage disorders, while neuroprotection was associated with proteins of the WAVE regulatory complex controlling actin cytoskeleton dynamics. Elevated ganglioside GM2 activator (GM2A) associates with reductions in both NI and MEA activity, and cell-derived GM2A alone is sufficient to induce a loss of neurite integrity and a reduction in neuronal firing.

**Conclusions:**

The techniques and data herein introduce a system for modeling neuronal vulnerability in response to factors in the human brain and provide insights into proteins potentially contributing to AD pathogenesis.

**Supplementary Information:**

The online version contains supplementary material available at 10.1186/s13024-022-00558-4.

## Background

Alzheimer’s Disease (AD) is the most common cause of age-related dementia and its prevalence is expected to grow in the coming years, with estimates as high as 100 million sufferers worldwide by 2050 [1]. From the original description of AD by Alois Alzheimer in 1906, the pathology was described as the co-occurrence of extracellular plaques and intracellular neurofibrillary tangles (NFTs), subsequently shown to be composed of amyloid beta (Aβ) and the microtubule-associated protein tau, respectively [2]. How these (and other) aspects of AD pathology are related to neurotoxicity remains an area of ongoing research. The ‘Amyloid Cascade’ hypothesis postulates that Aβ production and aggregation trigger a cascade of excessive tau phosphorylation and the formation of NFTs, reduction in synapses, and neurodegeneration [2, 3]. Findings that Aβ deposition within the brain begins decades prior to the onset of symptoms and that Aβ accumulates to high levels in some cognitively unimpaired individuals have suggested that other factors are driving neurotoxicity in addition to or in concert with Aβ.

Attempting to study any disease or biological process in model systems always comes with inherent compromises. The system must be sufficiently complex to mimic multiple facets influencing the in vivo physiology of the disease state, yet the system must be sufficiently reductionist to i) have clearly defined end-points, ii) be executable within reasonable time frames, and iii) allow for parallel testing of multiple perturbations. Therefore, in devising a strategy for modeling disease processes, one must contend with these confounding challenges: a trade-off between complexity and reductionism and the ability to robustly detect subtle changes. Here, we chose both structural (neurite integrity) and functional (spontaneous activity) measures for assaying the effects of human brain factors on neuronal cultures.

The loss of synapses and disruptions of neuronal structural integrity have long been established as the best correlates to the clinical manifestations of AD, more highly correlated with cognitive decline than amyloid burden [4, 5]. It has recently been shown that it is possible to monitor changes to neuronal structures in a dynamic fashion in vitro in response to exposure to aqueous-soluble brain extracts using a live-cell, morphological neurite integrity assay [6]. Here, we employed this system to assay across brain tissue samples from individuals that span the clinical and neuropathological spectrum of AD. To complement this system, we developed a new methodology that employs multi-electrode arrays (MEAs) for assaying the influence of human brain extracts on neuronal activity. MEAs are instruments that monitor spontaneous electrophysiological activity within in vitro neuronal cultures (reviewed in Negri et al., 2020 [7]). By monitoring the voltage potentials generated endogenously by firing neurons, these instruments do not require fluorescent dyes or exogenously expressed fluorescent proteins. Additionally, MEAs can simultaneously record across dozens to hundreds of channels in a less labor-intensive manner than the single channel recordings of conventional electrophysiological techniques such as patch-clamping or sharp-electrode recording. Moreover, MEA recordings are non-destructive to the cultures allowing for repeated recordings over time.

In this study, we find that levels of specific Aβ and tau peptides present in human brain tissue significantly associate with reduced spontaneous firing assay and/or reduced neurite integrity. However, neither alone are sufficient to explain the effects observed in these assays in response to the panel of brain tissue extracts tested. Rather, we find that the protein composition across the TBS-soluble brain tissue extracts is highly complex and heterogeneous, and it is likely that multiple neurotoxic and neuroprotective proteins act in concert to determine the consequences of the brain extracts in these assays. We identify proteins associated with effect size in each assay and find that ganglioside GM2 activator (GM2A) protein levels are associated with neurotoxic effects in both assays. In addition, GM2A levels are elevated in AD brain tissue in our cohort and are elevated in AD cerebrospinal fluid (CSF) in a cohort of 384 individuals (202 control vs. 182 AD). GM2A is a lipid transport protein that binds to and stimulates ganglioside GM2 degradation, and mutations in this gene result in GM2 gangliosidosis, AB variant [8]. We validate the neurotoxic effects of GM2A by showing that cell-derived GM2A is sufficient to induce an impairment of neurite integrity and reduction in mean firing rate in the absence of brain extract. The assays developed here provide a system for modeling neuronal vulnerability in response to factors present in the aged human brain, and the data acquired from these assays may provide insights into proteins that are mediating these effects.

## Methods

### Tissue culture

Preparation of MEA plates: 96-well multi-electrode plates (Axion Biosystems) were coated with poly-Ornithine (poly-O, Sigma Aldrich) and laminin (Sigma Aldrich), and Matrigel (Corning Life Sciences). Briefly, a day prior to establishment of cultures, poly-O:laminin solution (20 μg/mL poly-O and 5 μg/mL laminin in PBS) was added to the MEA plates in a volume of 60 μL per well, and incubated overnight at 37﻿°C. The day of culture plating, the poly-O:laminin solution was aspirated, and the plates were washed once with PBS. Matrigel was reconstituted 1:20 in ice-cold Dulbecco’s Modified Eagle’s Media (DMEM) culture media (Gibco) without added serum or antibiotics and passed through a 40 μm strainer (BD Falcon). The Matrigel solution was added to the MEA plates in a volume of 60 μL per well and incubated for at least 1 hr. at 37 ﻿°C.

Primary cortical cultures were established from either E18 Sprague Dawley rats (Charles River Laboratories) or E18 C57BL/6 mice (Charles River Laboratories). Dams were sacrificed by CO_2_ euthanasia under a protocol approved by IACUC of Brigham and Women’s Hospital. Dissection of complete cortex from pups was performed in ice-cold HBSS (Gibco) under a dissecting microscope (Zeiss). The dissected cortices were suspended in 0.25% trypsin-EDTA (Gibco) for 10 min at 37 °C, the excess trypsin-EDTA solution was then aspirated. The tissue was then triturated in Complete DMEM: DMEM culture media (Gibco) supplement with 5% fetal calf serum (Lonza) and 1X penicillin/streptomycin/L-glutamine (Gibco); with a 10 mL serological pipette before being passed through a 100 μm strainer (BD Falcon). Counts of the cell suspension were taken in triplicate, and the cell suspension was back diluted to 1.5 × 10^6^ cells/mL in Complete DMEM. The cell suspension was added to the MEA plates in a volume of 50 μL for a plating 452 concentration of 7.5 × 10^4^ cells/well or 2.4 × 10^5^ cells/cm^2^. Plates were then placed in a tissue culture incubator (37﻿°C, 95% humidity, 5% CO_2_) for 4 hrs to allow for cells to attach to the culture surface. After the 4-hr incubation, 150 μL of BrainPhys Media (1X BrainPhys culture media with SM-1 neuronal supplement [StemCell Technologies]) was added to each well. The cultures were maintained in a tissue culture incubator in BrainPhys Media, with semi-weekly half-volume media changes.

Human iPSC-derived neurons (iNs) were established from the YZ1 1.4 cell line using a Neurogenin-2 induction differentiation protocol [9, 10]. Cells were plated at Day 4 following the iN differentiation protocol at a density of 3000 or 5000 cells per well and grown in monoculture on 96-well glass-bottom plates (μClear, Greiner Bio-One) coated with Matrigel substrate (Corning) and grown until to Day 21 post-induction or longer depending on the treatment with brain extracts or cell-derived lysates.

### MEA recording

All MEA recordings were performed using a Maestro multi-well MEA recorder (Axion Biosystems). During recordings, plates were kept on a heated stage maintained at 37 ﻿°C and ventilated with a mixture of 5% CO_2_:95% air (Airgas) at a rate of 1 cubic foot per hr. To prevent evaporation of liquid within wells by convection and condensation on the underside of the plate lid, the MEA plate was covered with an air-activated oxidizing iron heater (HotHands) placed on top of an aluminum plate cut to size, for even dispersal of heat. Voltage potentials within wells were simultaneously recorded across 768 channels (8 electrodes per well in a 96-well plate) at a sampling frequency of 12 kHz using AxIS acquisition software version 2.4 (Axion Biosystems). The raw voltage recordings were subjected to a Butterworth filter of 200 Hz - 2.5 kHz, and neuronal firing events (spikes) were detected when the voltage exceeded a “crossing threshold” set at 5.5 standard deviations (SDs) away from the root mean squared (RMS) of the background potential calculated over a 10 ms moving window. All recordings were performed for 30 min unless otherwise specified.

### MEA analysis

Raw voltage, timestamp, and value of crossing threshold for each spike event were extracted from the .spk files of MEA recordings produced AxIS acquisition software, using custom MatLab scripts (MathWorks) using extractor functions provided with AxIS version 2.4. Following extraction of the raw recording data, all analyses and simulations were performed using the R statistical programming language (R Core Team 2017). All figures were generated using the R ggplot2 and accompanying ggpubr libraries.

Mean-firing rate calculation: To remove spurious spike events arising from by ‘high-noise’ electrodes, an upper limit to the crossing threshold was established by examining the crossing threshold (μV) for all spike events detected and calculated the value corresponding to 3 SDs greater than the mean crossing threshold, all events detected at a crossing threshold greater than this upper limit were excluded from the analysis. The mean firing rate (MFR, Hz) was calculated as the ratio of the total number of spikes record, n, and the duration of recording in seconds, s, MFR = n/s. The log transform of the MFR was calculated as log_10_[(n + 1)/s], to account for instances of *n* = 0, the log of which is undefined.

Treatment group assignment: A pool of *active* arrays from a multi-well MEAs is established by selecting those arrays that are no more than 2 SDs below the median of the sample set. A panel of *i* possible treatment assignments is generated by randomly assigning arrays to treatment groups *g*, each with *n* members. For the purposes of this study, *i* = 10^4^. For each instance of *i*, a one-way ANOVA was performed, assessing log10Hz as a function of *g*. The instance of *i* resulting in the lowest value of the F-statistic, was used as the treatment group assignment.

Power calculation: A dataset of 30-minute recordings of 1272 unique, untreated MEAs was taken to represent spontaneous firing activity log_10_ Hz at time (t_0_), this was defined as vector A. The correlation coefficient between the firing frequencies of a population MEAs recorded at separate times was estimated to be ρ = 0.8, based on the repeated recordings of multiple culture preparations at Day 20 and Day 21. To simulate the expected variance in firing frequencies between recordings, a second vector *B* was calculated by the formula:

$$B=\rho A+{A}^{\perp}\sqrt{1-{\rho}^2}$$

Where *A*^⊥^ represents the residuals of a linear regression between A and a sample of equal length drawn from a random normal distribution. The resulting value of B was examined to confirm that:

$$cor\left(\ A\to, B\to \right)=\rho$$

*B* is then taken to represent spontaneous firing activity log_10_Hz at time (t_1_). For each simulated experiment, control and treatment groups were generated by drawing the paired log_10_Hz_t0_ and log_10_Hz_t1_ values for random arrays for sample sizes ranging 3–16. The log_10_Hz_t1_ values within the *treatment* group were offset by effect sizes ranging from 0.1–2.0 log_10_Hz. 5000 iterations were performed for each sample size:effect size pairing, for a total of 1.4 × 10^6^ total simulated treatments. For each iteration, an ANCOVA was performed by fitting an ANOVA model to linear regression for log_10_ Hz as a function of group (*control*/*treatment*) and time (*pre*/*post*) allowing for interaction between the group and time variables. The coefficients for the model were compared using Tukey’s test for honest significant difference, and the *p*-value for the comparison of *control* verse *treatment* at time t_1_ was extracted. The linear regression, fitting of the ANOVA, and Tukey’s test were performed using the lm, aov, and TukeyHSD functions within the base R stats library. Power was calculated as the proportion of iterations within each sample size:effect size pairing for which the difference between *control* and *treatment* was calculated to have a *p* < 0.05. The simulation was performed on the O2 high performance computing cluster (Research Computing Group, Harvard Medical School).

### Neurite integrity assay

Human iNs of glutamatergic cortical fate were differentiated for 21 days before the assay. Cells were imaged every 2 or 4 hours using an IncuCyte system (Sartorius) and treated with brain extract or cell-derived lysates. Neurite integrity was quantified using the IncuCyte NeuroTrack Software Module (Sartorius). Total neurite length per well were normalized to the values from the first 6 hours of imaging. Additional details relating to this assay were previously published [6].

### Brain extract preparation

All work was performed following IRB review and approval through Partners/BWH IRB (2016P000867).

Human brain material was obtained from 1) the neuropathology core facility at Massachusetts General Hospital, 2) Rush University Medical Center, and 3) Albany Medical Center. Tris Buffered Saline (TBS: 20 mM Tris-HCl, 150 mM NaCl, pH 7.4) brain extracts were prepared using the method previously described [11]. Briefly, the tissue was dissected to isolate the gray matter, which was homogenized in ice-cold TBS at a ratio of 1:4 tissue weight to buffer volume within a Dounce homogenizer. This suspension was then subject to ultra-centrifugation at 1.75 × 10^5^ x *g* in a TL100 centrifuge (Beckman Coulter) to pellet cellular debris. The supernatant was place in a 2-kDa dialysis cassette (Slide-A-Lyzer, Thermo Fisher) and dialyzed 1:10^4^ (volume:volume) in artificial cerebrospinal fluid (aCSF: 124 mM NaCl, 2.5 mM KCl, 2.0 mM MgSO_4_, 1.25 mM KH_2_PO_4_, 26 mM NaHCO_3_, 10 mM glucose, 4 mM sucrose, 2.5 mM CaCl_2_). Following dialysis, the TBS brain extracts were aliquoted and stored at − 80 ℃. To treatment of cultures, the TBS brain extracts were ‘buffer-exchanged’ into culture media by placing extract in a 3-kDa centrifugation filter (Amicon Ultra, EMD Millipore) and performing a 90-min centrifugation at 3000 x *g* in an RC-5C centrifuge (Sorvall, Thermo Fisher). Following centrifugation, the retentate was reconstituted to 1X volume in BrainPhys Media with SM-1 neuronal supplement (StemCell Technologies). The protein concentration within the reconstituted extracts was determined by performing a bicinchoninic acid (BCA) assay (Thermo Fisher) on an 8-step, 2-fold dilution series of reconstituted extract and media to determine the proportion of protein contributed by the brain extract. The reconstituted extract was then back diluted in BrainPhys media to a concentration of 1 mg/mL brain extract protein and used for treatment of cultures.

### Amyloid-β and phosphorylated tau enzyme-linked immunosorbent assay (ELISA)

Quantification of Aβ within the dialyzed human brain extracts was performed using a multiplex ELISA for Aβ38, Aβ40, and Aβ42 (V-Plex Plus Aβ Panel, Mesoscale Discovery). Oligomeric Aβ (71A1 and 1C22) and Aβ37 were quantified as previously described [12–14]. The Aβ detected within the native extract was designated as *soluble* Aβ. In order to quantify the additional, aggregated Aβ, extracts were treated with 6 M guanidine hydrochloride (GuHCl) to denature the proteins in the mixture prior to performing the ELISA. Native extract samples were diluted 1:10, and GuHCl treated extracts were diluted 1:35, prior to being added to the ELISA plate. Aβ38 levels were below the detectable range of the assay for most samples and thus were not reported. ELISA of the dialyzed human brain extracts was performed for the quantification of pT181 (Mesoscale Discovery) [15] and pT217 (Liu et al., in submission).

### Immunocytochemistry

Rat primary cortical cultures were established in black-walled, glass bottom 96-well plates (μClear, Greiner Bio-One) with the same cell density and culture conditions as described for MEA experiments. Following a 4-day treatment with extract from AD patient, ALB01, or vehicle (media alone), the cultures were washed with ice cold PBS and fixed with 4% paraformaldehyde in PBS. Following fixation, the cells were permeabilized with 1% Triton X-100 in donkey serum (Jackson ImmunoResearch). The cells were probed with primary antibodies for tau (1:200, A0024, Dako), followed by anti-rabbit Cy3 secondary antibodies (Jackson ImmunoResearch) and 1 μg/mL DAPI (Thermo Fisher). Mouse anti-Vglut2 (Abcam, 1:1000) and rabbit anti-HOMER1 (Synaptic Systems, 1:1000) were used to quantify VGLUT2 and HOMER1 in aged (Day 21) iNs transduced with the lentivirus (Lenti-eGFP or Lenti-GM2A) at MOI = 5 on Day 5 or treated with TBS or AD brain extract (ALB01) from Day 17. Analysis was performed using Fiji (National Institutes of Health) by counting the number of puncta along each neuritic segment (~ 10 um), and 15 images were counted in each condition. Co-localization of puncta was defined by overlap within a circular area covering 0.52 um^2^. Images were acquired at 40X using an LSM710 confocal microscope (Zeiss).

### Proteomic analysis

Unlabeled proteomic analysis was performed on the TBS-soluble brain extracts following dialysis in aCSF. Liquid chromatography coupled to tandem mass spectrometry (LC-MS/MS) was performed by the laboratory of Dr. Nicholas Seyfried at Emory University using a NanoAcquity UHPLC (Waters) and Q-Exactive Plus mass spectrometer (Thermo Fisher) using methods previously reported [16, 17]. Label-free quantification (LFQ) of proteins was performed using the MaxQuant algorithm [18]. Variance stabilization was used to normalize LFQ values across samples using the R vsn library [19].

The samples of brain extracts were processed in 2 separate LC/MS-MS runs. Of the 43 individuals examined, samples of 20 individuals were analyzed exclusively in the first round, 16 were analyzed exclusively in the second round, and 7 were analyzed in both rounds. In order to account for non-biological variability introduced by sample preparation and instrument detection, the LFQ values were normalized using variance stabilization normalization (VSN) as recommended by Valikangas and colleagues [19, 20], common to most methods of normalization for proteomic data. Normalized LFQ values were then log2 transformed in order to account for the highly skewed distributions of raw LFQ values. Any residual effects of the separate analysis rounds were accounted for by calculating and removing the effect of run on each protein using linear regression.

A known short-coming of label-free proteomic analysis is the occurrence of failed or missed quantification of proteins, in particular amongst those proteins with lower expression [16, 21]. Instances in which the quantification of a given protein was missed in an excessive number of samples presents a challenge when attempting to make inferences about the effect of that protein on a particular phenotype. Therefore, any protein in which quantification was missed in at least 5 samples was excluded for the purposes of this analysis. This translates to a requirement that a given protein needed to be quantified in 39 of 43 individuals (90.6%).

### GM2A TBS-soluble lysate preparation

HEK293 cells cultured in DMEM (ATCC) with 10% Fetal Bovine Serum were transfected with GM2A cDNA-containing construct (pLenti-GM2A-mGFP, Origene) using Lipofectamine 3000 (Thermo Fisher) for 72 hr. following manufacturer’s protocol. HEK293 cells were washed with PBS and spun down at 200 x *g* for 5 min at room temperature. Cell pellets were lysed in 250 uL ice-cold TBS with protease inhibitor cocktail (cOmplete, Roche) and phosphatase inhibitor cocktail (PhosSTOP, Roche), followed by 3 cycles of freeze (in dry ice) and thaw (at 37 °C). Supernatant was collected after centrifugation at 10,000 x *g* in the centrifuge (5417R, Eppendorf) for 10 min at 4 °C.

### Lentiviral transduction

Lenti-eGFP (EX-EGFP-Lv236) and Lenti-GM2A (EX-Z5875-Lv236) were purchased from GeneCopoeia. Lentiviruses were packaged by Alstem with ultrahigh titers (10^9^) and used at the following concentrations: multiplicity of infection (MOI) = 1, 3, or 5 in 3 K cells/well for IncuCyte analyses and in 25 K cells/well for MEA.

### Western blot analysis

The brain extracts and cell lysates were separated by 4–12% Bis-Tris gels (Thermo Fisher), followed by transfer to nitrocellulose membrane using Criterion blotter system (Bio-Rad). Nitrocellulose membranes were incubated with Intercept blocking buffer (LI-COR) for 1 hr. at room temperature, followed by overnight incubation with rabbit anti-GM2A antibody (1:1000, Proteintech), mouse anti-GAPDH (1:10,000, Proteintech), rabbit anti-HOMER1 (Synaptic Systems, 1:1000), or mouse anti-Vglut2 (Abcam, 1:1000). Membranes were washed 3 times with TBST and incubated with fluorescent dye-conjugated secondary antibodies (anti-mouse or anti-rabbit, 1:10,000, LI-COR) for 1 hr. at room temperature. Membranes were washed 3 times with TBST and then 2 times with TBS, followed by scanning using an Odyssey Infrared Imaging System (LI-COR).

### Statistics

Spontaneous firing frequencies of cultures were assessed using the array mean-firing-rate during each 30-minute recording. For comparing the effects of treatment conditions within single experiments (e.g. *extract immuno-depletion*, *boiling*, *fractionation*), an ANCOVA was performed by fitting a linear regression for log_10_Hz as a function of treatment group and time allowing for interaction between the group and time variables, and treating time as the covariate. For comparing the effects of treatments across several experiments (e.g. *individual extract* or *diagnosis*), a linear mixed effect (LME) model was fit to the data. For the model estimating the effect of diagnosis, diagnosis and time were treated as fixed effects and individual, experiment, and array as nested random effects. For the model estimating the effect of individual exact, individual and time were treated as fixed effects, and experiment and array were treated as nested random effects. In both cases, interaction between the fixed effects (diagnosis/individual and time) was modeled, since the different diagnoses or individuals would be expected to have differing effects over time. Significance between the model coefficients from the ANCOVA and LME models was determined using a general linear hypothesis test. To estimate the effect of soluble and insoluble Aβ42 on firing frequency, a multivariant linear regression model was fit. The linear regressions were fit using the lm function within the base R stats library, while the LME were fit using the lme function within the nlme library (R Core Team 2017 [22, 23]). The general linear hypothesis tests were performed using the glht function within the multcomp library after generating a contrast matrix for the comparisons of interest, and multiple comparison were accounted for using the default single-step multivariant t comparison test.

Comparisons between group were performed using a one-way ANOVA followed by Tukey post-hoc test, unless otherwise specified. Comparing instances of diagnosis class across protein expression clusters was performed using Pearson’s Chi-squared test. In cases of multiple comparisons, adjusted *p*-values were calculated using the methods described by Holm (1979). All analysis was performed using the R statistical programming language. Figures and tables were generated using the R ggplot2, ggpubr, and kableExtra libraries [24–26].

Hierarchical Clustering: Agglomerative (bottom-up) hierarchical clustering was performed using complete linkage based on pairwise calculation of Spearman’s correlation between the vectors of protein expression for each individual extract [27]. Missing values in the LFQ matrix were imputed based on columns means, however this was only necessary for 0.8% of observations (646 of 79,163). Hierarchical clustering was performed using the hclust and cutree functions within the base R stats library.

Differential Expression: Differential expression analysis was performed using one-way ANOVA as described [28].

Correlation analysis: Spearman’s correlation (Spearman’s ρ) was calculated between the two numerical vectors representing *i* the assay metric (% neurite integrity or ∆Log10Hz) for the n individuals examined in each assay and *ii* the n protein expression values (normalized LFQ) for a given protein in those individuals. This calculation was repeated for each protein reliably detected across the panel of brain extracts tested in a given assay. In order to determine the probability of each observed correlation, the ρ value was converted to a z-score under a random normal distribution by applying Fisher’s transformation. Fisher’s transformation was applied using the FisherZ function within R DescTools library with modifications for Spearman’s ρ as shown below:

$$z=\sqrt{\frac{n-3}{1.06}}\times F\left(\rho \right)$$

## Results

### Overview of the study

Multiple studies have explored the proteomic landscape of the human brain, with the purpose of identifying proteins and pathways differentially altered in AD [16, 29–31]. While highly valuable, the impact of those studies has been limited by the availability of assays for measuring the functional consequences of differentially expressed proteins in the human brain. Therefore, a challenge in the field has been to disentangle protein changes that are driving synaptic loss and neurodegeneration from those that are indirect consequences of neuronal death. We aimed to identify the proteins present in the aged brain that may have neurotoxic or neuroprotective activities, using unbiased proteomics coupled to NI and MEA assays of structural and functional integrity of neuron.

**Fig. 1:**
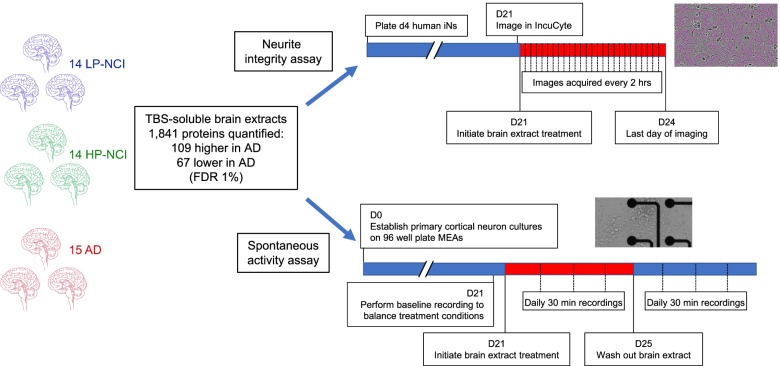
Overview of study. Brain tissue from the prefrontal cortex was acquired for 43 individuals, and TBS-soluble extracts prepared. Tissue was extensively dialyzed in BrainPhys media to remove small molecules, such as glutamate, present in the extracts. Extracts were analyzed by proteomic profiling, and 1841 proteins quantified. These extracts were used either in a neurite integrity assay and/or in an MEA-based spontaneous activity assay. Correlations were then calculated between proteins quantified via proteomics and activity in each assay

Brain tissue was acquired postmortem from 43 individuals, 14 with low AD neuropathology who were not cognitively impaired (LP-NCI), 14 with high AD neuropathology who were not cognitively impaired (HP-NCI), and 15 with clinical and pathological diagnoses of AD. Table 1 outlines distributions of sex, age at death, and postmortem interval (PMI) across brain samples. The mean age at death was 81 and was not significantly different across diagnostic categories. Tris buffered saline (TBS)-soluble extracts were prepared from the prefrontal cortex and analyzed by unbiased proteomics. The same TBS-soluble brain extracts also were used in either 1 or 2 assays of neuronal structure and function: an imaging-based morphological NI assay and/or an MEA-based spontaneous firing assay (limiting amounts of available brain tissue precluded the use of all extracts in both assays). Proteins identified were those associated with neurotoxicity or neuroprotection in each assay. An overview of the experimental design of the study is outlined in Fig. 1.

**Table 1.** Brain samples used in this study

**Brain ID**	**Category**	**Age**	**Sex**	**PMI**	**Assay**
BWH04	LP-NCI	53	M	ND	MEA
MGH2038	LP-NCI	78	F	6	MEA
MGH1887	LP-NCI	60	M	ND	MEA
MGH2018	LP-NCI	92	F	24	MEA
RM100	LP-NCI	88	M	9	NI
RM105	LP-NCI	75	F	3	NI
RM106	LP-NCI	91	F	15	NI
BWH02	LP-NCI	76	F	ND	MEA
MGH1837	LP-NCI	68	M	27	MEA
MGH2068	LP-NCI	79	F	9	MEA
RM101	LP-NCI	76	M	5	NI
RM102	LP-NCI	82	M	6	NI
RM103	LP-NCI	85	M	8	NI
RM104	LP-NCI	79	F	18	NI
RM207	HP-NCI	89	F	17	NI
RM205	HP-NCI	83	F	6	NI
RM208	HP-NCI	80	F	5	MEA / NI
MGH1846	HP-NCI	92	M	ND	MEA
RM201	HP-NCI	78	F	24	NI
RM206	HP-NCI	85	F	12	NI
RM200	HP-NCI	86	M	7	NI
BWH03	HP-NCI	77	M	ND	MEA
MGH1965	HP-NCI	76	F	48	MEA
BWH01	HP-NCI	76	F	ND	MEA
RM202	HP-NCI	88	F	12	NI
RM204	HP-NCI	82	F	8	NI
RM203	HP-NCI	86	F	13	NI
RM209	HP-NCI	87	F	15	NI
MGH1892	AD	67	M	28	MEA
RM304	AD	87	F	5	MEA / NI
MGH2031	AD	70	F	8	MEA
RM301	AD	81	M	12	NI
RM305	AD	87	F	10	MEA / NI
ALB01	AD	68	F	18	MEA / NI
MGH2037	AD	73	F	22	MEA
MGH2039	AD	74	M	24	MEA
RM303	AD	87	F	17	NI
RM306	AD	90	F	5	NI
RM302	AD	89	M	4	NI
RM307	AD	91	F	7	NI
RM308	AD	98	F	7	NI
MGH1168	AD	89	F	ND	NA
RM300	AD	85	M	5	NI

Postmortem prefrontal cortex was acquired from 43 individuals. Listed are category, age at death, postmortem interval (PMI), sex, and assay(s) in which the samples were used. Due to limited supply brain tissue for many of the samples, most were used in only 1 of the 2 assays.

*Abbreviations*: *LP-NCI* low pathology and non-cognitively impaired, *HP-NCI* high pathology and non-cognitively impaired, *AD* Alzheimer’s disease, *ND* not determined/not known, *M* male, *F* female, *MEA* multi-electrode array spontaneous activity assay, *NI* neurite integrity assay.

### Differentially expressed proteins in the TBS-soluble AD brain proteome

**Fig. 2:**
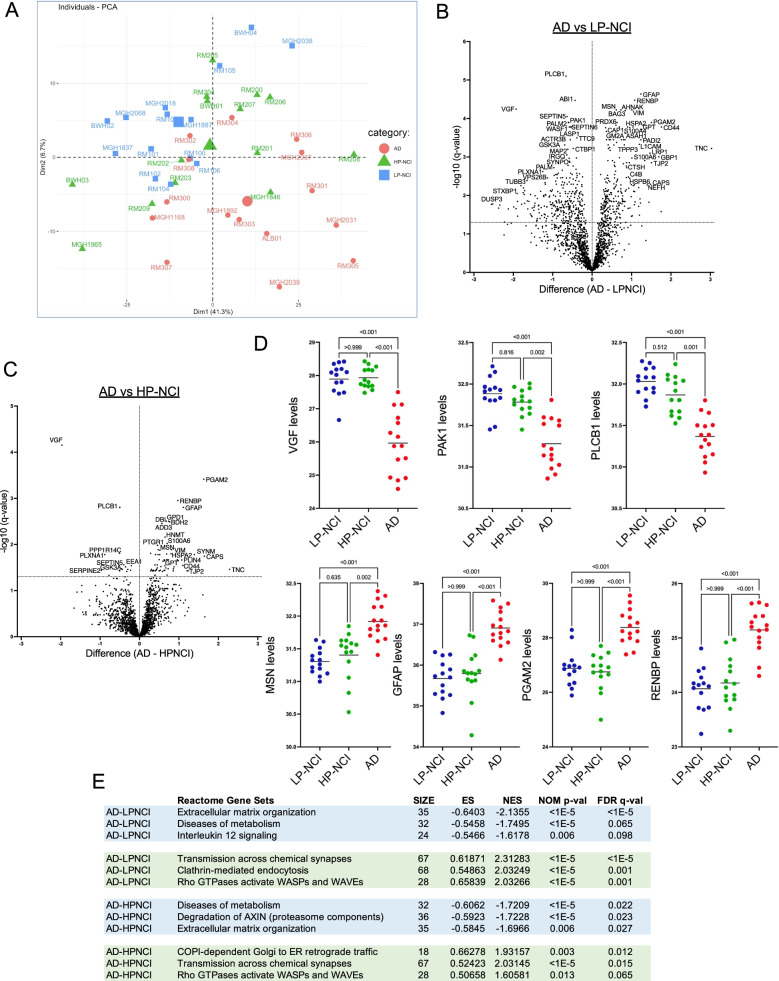
Differentially expressed proteins in the TBS-soluble AD brain proteome. **A** PCA plot of brain tissue samples using proteomic data. AD TBS-soluble proteomes are heterogeneous within categories, with overlap across categories. **B**, **C** Differential expression of individual proteins was analyzed between AD vs. LP-NCI (B) and AD vs. HP-NCI (C). Many more proteins were differentially expressed in the LP-NCI vs. AD comparison than in the HP-NCI vs. AD comparison. Unpaired t-test with Welch correction, two-stage step-up, dotted line at q = 0.05. **D** Scatter plots of top DEPs across categories. Units are normalized abundance ratios. One-way ANOVA with Dunn’s multiple comparisons test, *p*-values as listed. **E** Top hits in the REACTOME database for GSEA of analyses in B&C. Size = number of components in that gene set, ES = enrichment score, NES = normalized enrichment score

The proteome in each of the 43 brain extracts was examined by unbiased proteomic analysis using liquid chromatography/tandem mass-spectroscopy (LC/MS-MS), followed by label-free quantification (LFQ) to detect 1841 proteins across the brain samples [16, 18]. Principal components analysis (PCA) demonstrates that AD samples tend to cluster together and away from LP-NCI samples, while HP-NCI samples intermix with each. This may be because the category of HP-NCI is likely to include both those individuals who have sustained, strong cognitive resilience even in the presence of high amyloid pathology and individuals who may have developed AD had they lived longer. While there is some separation between these categories in PCA space (Fig. 2A), there is clearly a lot of overlap across categories in the proteome-wide profile.

An analysis of differentially expressed proteins (DEPs) by category revealed 601 DEPs between AD vs. LPNCI and 80 DEPs between AD and HP-NCI (FDR < 0.05; Fig. 2B, C). Importantly, these proteome profiles may show substantial differences compared to profiles previously reported from similarly aged individuals [16, 30, 31], because here we are only measuring the TBS-soluble proteome, while most other studies reported the urea-soluble proteome. The top DEP between AD and NCI was VGF, which was lower in the AD brain relative to both LP-NCI and HP-NCI brain (Fig. 2B-E). VGF levels in the brain have previously been reported to significantly associate with AD [32]. In addition to VGF, some DEPs identified here validate findings in prior studies, such as MSN and GFAP [33, 34], while other DEPs have not previously been linked to AD, such as PGAM2 and RENBP (Fig. 2D). Gene set enrichment analysis (GSEA) of DEPs using the REACTOME database showed significant associations (False Discovery Rate [FDR] < 0.05) with specific terms including extracellular matrix organization, transmission across chemical synapses, Rho GTPases activate WASPs and WAVEs, and diseases of metabolism (Fig. 2E).

### Assessing effects of human brain extracts on spontaneous neuronal firing using multi-electrode arrays

The generation of action potentials in neuronal cultures can be assessed using a multi-electrode array (MEA) based spontaneous firing assay. We used MEA recordings to assess the effect of TBS-soluble brain extracts on spontaneous firing of primary rat neurons in dense, mixed-cortical cultures (treatments on Day 21 of culture).

**Fig. 3:**
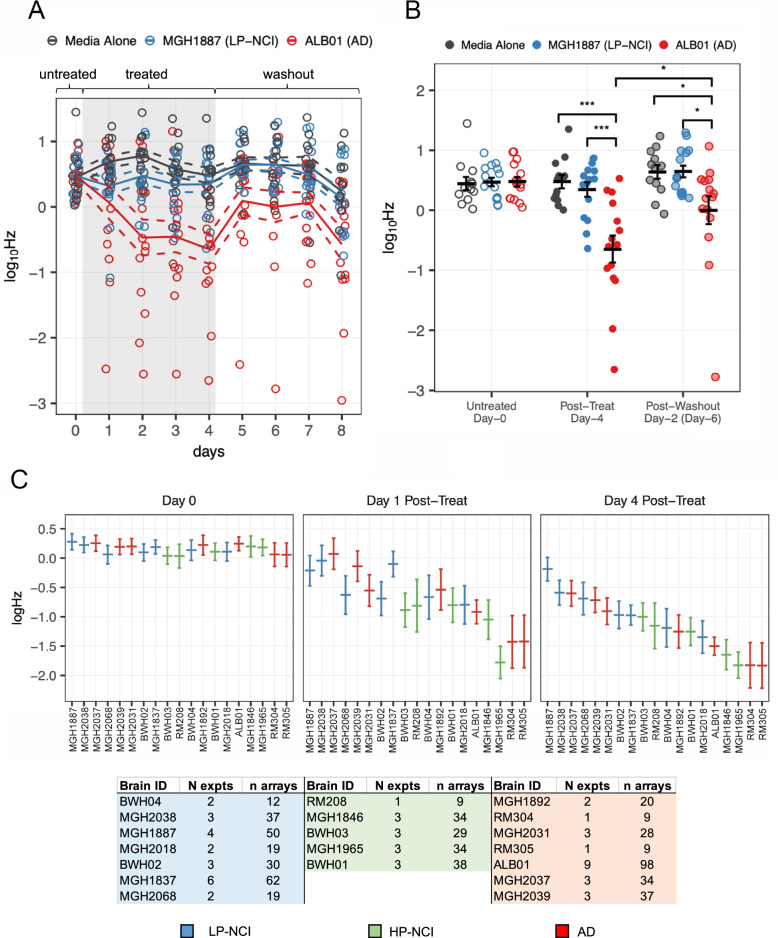
Effect of human brain extract on spontaneous firing of cultured cortical neurons. **A** Time course of firing activity within rat cortical cultures prior to, during, and following treatment with human brain extract from 2 individuals. Each dot represents a single multi-electrode array (8 electrodes per array) during each recording. Solid line tracks the mean firing frequency per condition at each recording, and dashed lines indicate mean ﻿ ± SEM. **B** Comparison of firing frequencies across treatment conditions prior to, 4-days post-treatment, and 2-days following washout. Error bars represent mean ﻿ ± SEM. Significant differences between conditions indicated by brackets, *, *p* < 0.05, ***, *p* < 0.001. **C** Effects on spontaneous firing elicited across a panel of human brain extracts. An estimated effect on spontaneous firing of individual rat neurons at baseline (Day 0), 1-day, and 4-days following treatment with human brain extracts. Error bars represent mean ﻿ ± SEM as predicted by linear-mixed effect model based on treatment data from 19 individuals across 1 to 9 replicate experiments per individual. Numbers below indicate number of experiments (“N”) and number of independent arrays (“n”) tested for each brain sample

TBS-soluble brain extracts were dialyzed 1:10,000 and buffer exchanged into BrainPhys media after centrifugation over a 3-kDa cutoff column (StemCell Technologies). These brain extracts were normalized by protein concentration across all samples, such that extracts contained 1 mg/mL of brain derived total protein. First, to characterize the experimental system and optimize conditions, extracts were prepared from tissue samples of an unaffected, non-cognitively impaired control individual (MGH1887) and an AD diagnosed individual (ALB01). These extracts were diluted 1:1 in BrainPhys media, and then used to treat Day 21 primary rat cortical cultures grown on MEAs. While similar AD brain extracts have been shown to inhibit LTP within rodent brain organotypic slice cultures within minutes to hours [11, 35, 36], the maximal effect on spontaneous firing induced from the AD brain extract emerged over 4 days (Fig. 3A). After 4 days of treatment with AD brain extract, we observed significantly decreased spontaneous firing by approximately an order of magnitude compared to those treated with control brain extract (− 0.993 ∆log10Hz, *p* = 2.07 × 10^− 5^; Fig. 3A, B).

After the removal and washout of the brain extract treatment, there was a partial recovery in spontaneous firing within the AD brain extract treated cultures, peaking 2-days following the exchange of extract-containing media with fresh, untreated culture media (Fig. 3B). While the firing activity was still significantly diminished in the AD brain extract-treated culture 2-days following the washout of the treatment, compared to the activity in the cultures previously treated with control brain extract (0.646 ∆log10Hz, *p* = 0.0158), the extent of recovery within the AD brain extract treated cultures was itself significant (0.648 ∆log10Hz, *p* = 0.0152). This recovery in spontaneous firing activity is consistent with previous reports that synaptic structures within cultured neurons are able to regenerate following exposure to recombinant Aβ species [37]. Further, examination of the cultures after 4-days of treatment by immunocytochemistry showed that the integrity of cells within the AD brain extract treatment condition was largely intact (Supplemental Fig. 1A).

Subsequent experiments showed that the potency of the effects on spontaneous firing induced by the AD brain extract extended over a narrow range (Supplemental Fig. 1B). This was determined by treating cultures with brain extract that had been further diluted in culture media using a half-log (1:3) dilution series. Within a 10-fold dilution of the AD brain extract, the difference in firing between the AD brain extract treated cultures, and those treated control brain extract was completely attenuated.

While the brain extracts were dialyzed, it is possible that residual pharmacological agents, metabolites, or neurotransmitters could be responsible for the inhibition of firing. In order to determine whether the effect was likely due to proteinaceous species within the extracts, cultures were treated with brain extracts from two AD individuals (ALB01 & MGH1892) in either their native form or following denaturation by boiling at 100 °C for five minutes. After a 4-day treatment, activity remained significantly higher in the cultures treated with boiled extract compared to those treated with native extract (ALB01: ∆log10Hz = 1.04, *p* = 1.67 × 10^− 6^; MGH1892: ∆log10Hz = 0.804, *p* = 0.0364; Supplemental Fig. 1C).

To understand whether the effects on spontaneous activity were representative of AD and unaffected patient populations as a whole, these experiments were extended to test a panel of human brain extracts from 19 individuals (Fig. 3C, see also Table 1). The individuals within this panel were designated to 1 of 3 classes defined by neuropathology and clinical diagnosis (*n* = 7 LP-NCI, *n* = 5 HP-NCI, and n = 7 AD). The primary samples within this panel were acquired periodically over a period of 2 years, and the amount of tissue obtained from each individual ranged from a few grams to an entire hemisphere of the brain. The consequence of this is that it was impossible to test all subjects in all experiments in a head-to-head fashion or to test all individuals the same number of times given the disparities in the material available. In total, the brain extracts from the 19 individuals have been tested across 22 experiments.

To make inferences about the effect of each brain extract on spontaneous firing, we used a linear mixed effect model [23] comprised of data from all experiments to estimate the firing frequencies observed in the treated cultures as function of individual extract and time. Interestingly, despite the brain extracts representing three distinct patient populations, the degree of reduction of firing is continuous across the panel of individuals. AD and HP-NCI individuals tended to induce a greater decrease in firing activity than those of extracts of LP-NCI individuals, however, the differences do not reach the significance threshold of *p* < 0.05 (LP-NCI vs. AD *p* = 0.185; LP-NCI vs. HP-NCI *p* = 0.138).

### Both HP-NCI and AD brain extracts induce degeneration of neurites compared to LP-NCI brain extracts

**Fig. 4:**
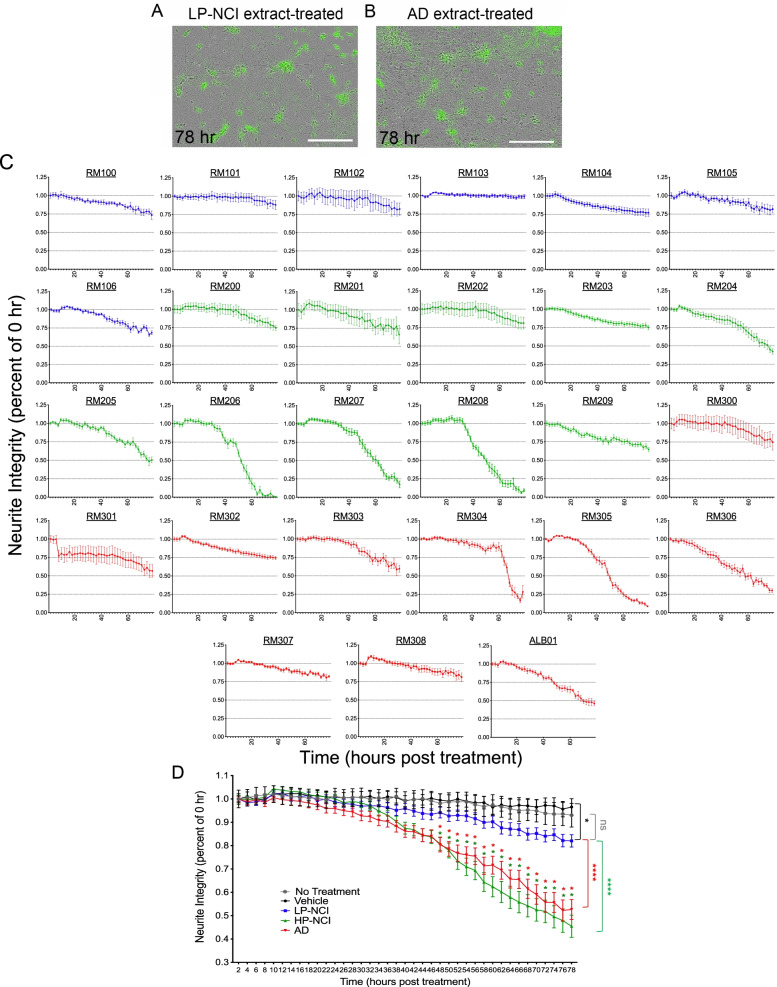
Influence of human brain extracts on neurite integrity in vitro. **A, B** Representative images of human iPSC-derived neurons (iNs) treated with an LP-NCI brain extract (A) and neurons treated with AD brain extract (B) at 72 hr. post-treatment. Scale bars = 200 μm. **C** Quantification of data for each individual brain extract over 78 hours. Data show mean ﻿ ± SEM; *n* = 6 for each brain extract. **D** Estimated neurite integrity following 72-hr treatment by diagnosis class, combining all data from (C). *N* = 36 for vehicle-treated wells, and *n* = 36 for no treatment condition. Neurite integrity in human iNs normalized to baseline (time 0), examined at 2-hr intervals over 78-hr treatment with human brain extracts. One-way ANOVA with Tukey’s multiple comparisons test, comparing LP-NCI, HP-NCI, and AD-extract treated conditions. Significance is shown relative to the LP-NCI extract-treated condition. *, adjusted *p* < 0.005; **** *p* < 0.0001

A recent study established and characterized a live-cell, morphological neurite integrity (NI) assay to monitor changes to neuronal processes in a dynamic fashion in response to exposure to aqueous-soluble brain extracts [6]. Using the same experimental procedure as reported by Jin and colleagues [6], neurite integrity in cultured human iPSC-derived neurons (iNs) was monitored at 2-hour intervals for 78 hours following treatment with TBS-soluble human brain extracts from 27 individuals (Fig. 4). At the end of the treatment time, neurites treated with some brain extracts remained intact (Fig. 4A, C) while cultures treated with other brain extracts became fragmented (Fig. 4B, C).

Human iNs showed a continuous response across the brain extract treatments, with some extracts causing negligible effect and others causing a near complete atrophy of neurites at the end of the recording (Fig. 4C). Examining the effects on neurites in the context of diagnosis shows that extracts from AD and HP-NCI individuals induced significantly stronger effects than LP-NCI brain extracts (Fig. 4D) (78-hr-timepoint, ANOVA with Dunnett’s multiple comparisons test: HP-NCI *p* < 0.0001; AD *p* < 0.0001). However, there was no significant difference between populations of HP-NCI and AD brain extracts.

### Levels of specific Aβ and tau peptides in brain extracts correlate with effect size in the MEA and NI assays

**Fig. 5:**
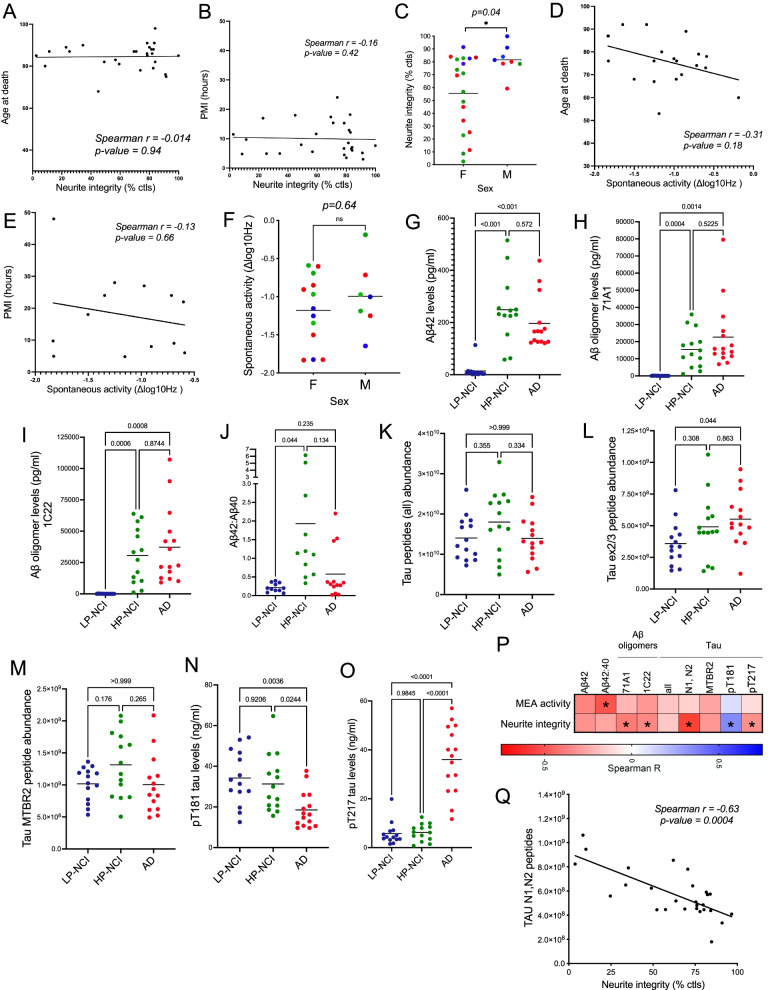
Associations of Aβ and tau peptides with MEA and neurite integrity. **A**-**F** Effect size on neurite integrity and spontaneous activity was examined as a function of age at death (A&D), postmortem interval (B&E), and sex (C&F). Data points in C&F are colored by category: LP-NCI (blue), HP-NCI (green), and AD (red). *P*-values were calculated by Spearman correlations (A-B&D-E) or Mann-Whitney test (C&F). **G-J** Aβ levels were measured by ELISA in TBS-soluble brain extracts and compared across categories. Aβ42 (G) and Aβ42:40 (H) were measured by 6E10 MSD ELISA, and Aβ oligomers were measured using two different ELISAs: both used 3D6 for detection and for capture antibody one used 71A1 and the other used 1C22. ANOVA with Dunnett T3 multiple comparisons test, *p*-values as listed. **K-O** Tau peptides were measured using mass spectrometry and ELISA and compared across categories. Scatter plots show the relative abundance of all tau peptides (K), tau peptides of the N1 and N2 domains “N1,N2” (L), tau peptides of the second MTBR, “MTBR2” (M), and tau phosphorylated at T181 (pT181) and T217 (pT217) (N-O). One-way ANOVA with Dunnett T3 multiple comparisons test, *p*-values as listed. **P** Heatmap of Spearman correlation coefficients calculated between the measures in (G-M) and effect size in the MEA and neurite integrity assays. *, *p* < 0.05. **Q** Graph of the strongest association between tau peptides with the N-terminal inserts and neurite integrity. *p* = 0.0004

Having tested brain extracts from 43 individuals in two assays, we next aimed to identify candidate factors that may be contributing to the effects observed in each assay. PMI and age at death were not significantly associated with effect size in either assay (Fig. 5A, B, D, E). Sex of the brain tissue donor was associated with effect-size in the NI assay (t-test, *p* = 0.04), but not in the MEA assay (t-test, *p* = 0.64). While intriguing, the distribution across categories was not the same between males and females, with fewer males in the LP-NCI category (Fig. 5C, F). Thus, any interpretations of this potential effect of sex must be made with caution.

The AD brain accumulates Aβ and tau in plaques and tangles, respectively. Previous studies have suggested that certain forms of soluble Aβ and tau are neurotoxic, and some AD brains induce neurite degeneration in vitro and inhibit long-term potentiation (LTP) in an Aβ-dependent manner [11, 35]. Therefore, we first measured Aβ and tau levels in this collection of brain extracts. Aβ was measured via ELISA before and after treating the extracts with the denaturing agent guanidine hydrochloride (GuHCl), which allows for epitope exposure within Aβ. As shown in Supplemental Table 1, the concentration of aqueous soluble Aβ42 is in the range of hundreds of pg/mL within the TBS-soluble extracts prepared from these individuals. These values are similar to the concentrations of Aβ42 in CSF observed within a similarly aged population [38]. Additionally, the aqueous insoluble Aβ42 concentration, especially amongst the HP-NCI and AD individuals, falls within the low ng/mL range. This mirrors the concentration of Aβ42 found within the tissue of symptomatic AD individuals processed using a denaturing extraction protocol [39]. It has been previously suggested that it is the oligomeric form of Aβ rather than the monomeric form that is the neurotoxic species present in the brain [11, 36, 40]. Therefore, we measured Aβ oligomer levels using two oligomer-specific ELISA platforms [12]. Both Aβ42 levels and oligomeric Aβ levels were significantly higher in AD and HP-NCI TBS-soluble brain extracts compared to LP-NCI TBS-soluble brain extracts, and not significantly different from one another (Fig. 5G-I). The ratio of Aβ42:Aβ40 is consistently elevated with hundreds of familial AD mutations. Here, Aβ42:Aβ40 levels were significantly higher in HP-NCI extracts compared to both LP-NCI and AD extracts in this brain tissues that were not from FAD carriers (Fig. 5J). This was a surprising observation, as our previous analyses of another cohort of TBS-soluble brain tissue showed significant elevation of Aβ42:Aβ40 in both HP-NCI and AD compared to LP-NCI [14]. Here, in addition to being a separate cohort of brains, there also were differences in the preparation of the brain samples including the addition of the 3 kDa molecular weight cutoff filter and the dialysis step. In addition to Aβ, tau was quantified via mass spectrometry. Overall tau levels were unchanged across categories (Fig. 5K). Tau is alternatively spliced in the brain to produce 6 isoforms which contain either 3 or 4 microtubule-binding repeats (“MTBRs”) and 0, 1, or 2 N-terminal inserts (“N”). Here, we identified and quantified peptides encoding the N inserts (1 N, 2 N) and peptides encoding the alternatively spliced MTBR (the second MTBR, MTBR2). Overall tau and MTBR2-containing tau levels were not significantly different across categories, while levels of peptides encoding the N inserts were significantly higher in the AD brain extracts compared to LP-NCI (Fig. 5K-M).

We next examined whether these Aβ and/or tau measures in the TBS-soluble extracts are associated with effect size in either the NI assay or the MEA assay by calculating Spearman correlation coefficients. Higher levels of each of these measures of Aβ and tau trended toward stronger effect size in each assay (Fig. 5P). The ratio of long to short Aβ (42:40) was associated with effect size in the MEA assay (Spearman r = − 0.58, *p* = 0.017). In the NI assay, both measures of oligomeric Aβ were significantly associated with effect size (71A1: Spearman r = − 0.46, *p* = 0.016; 1C22: Spearman r = − 0.42, *p* = 0.03). Two phosphorylated tau isoforms, pT181 and pT217 used as biomarkers for Alzheimer’s disease in CSF [41, 42], also were significantly associated with effect size (pT181: Spearman r = 0.418, *p* = 0.03; pT217: Spearman r = − 0.389, *p* = 0.045) (Fig. 5N-P). Intriguingly, as pT217 went up, neurite integrity went down, and pT181 showed the opposite association, and the meaning underlying this difference will be a subject of future studies. Interestingly, tau peptides with N-terminal inserts showed the strongest association with effect size in the NI assay (Spearman r = − 0.63; *p* = 0.0004; Fig. 5Q).

### Identification of proteins in the human brain associated with neurite degeneration and loss of spontaneous activity

**Fig. 6:**
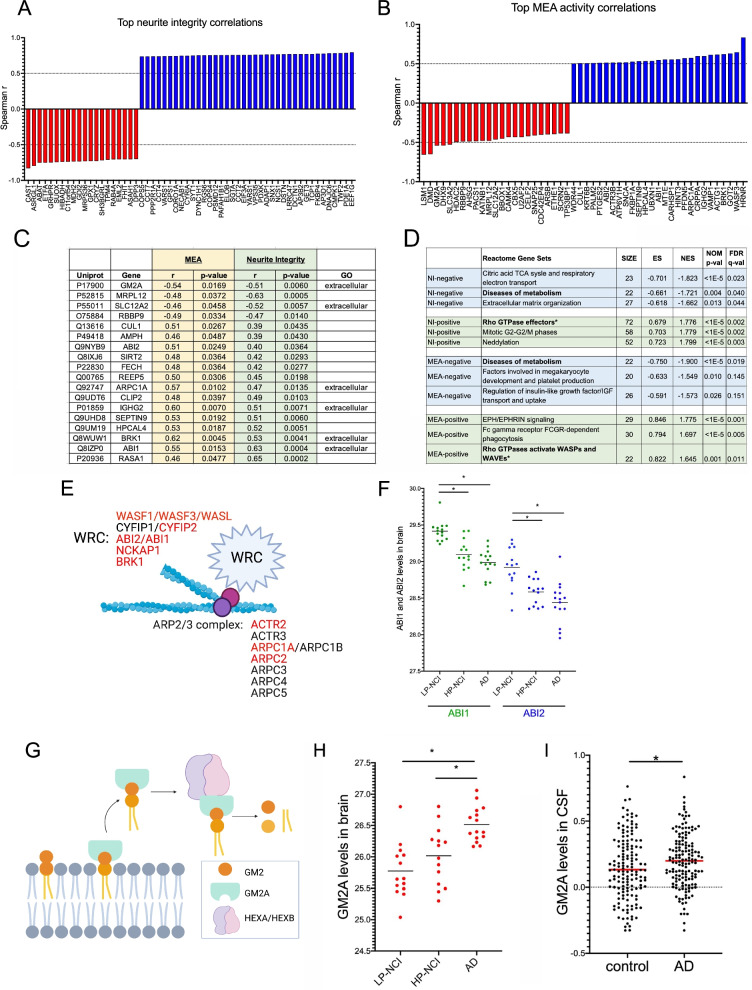
Associations between individual proteins across brain samples and estimated effect size in assays of neuronal structure and function. **A**, **B** Waterfall plots showing top associated proteins in the neurite integrity (A) and spontaneous activity (B) assays, calculated with the Spearman correlation coefficient. **C** Table of proteins meeting a *p*-value of < 0.05 across both assays. Top associations in both assays included ABI1, which showed a protective association in the assay, and GM2A which showed a neurotoxic association. **D** GSEA analyses using the REACTOME database for associations with the MEA and NI assays identify Diseases of metabolism and Effectors of Rho GTPase signaling/WASP and WAVE complexes with effect size in both assays. **E** Schematic of WAVE regulatory complex (WRC) and ARP complexes, gene symbols in red were identified as leading-edge genes in the GSEA analysis in (D). **F** Scatter plot of ABI1/ABI2 across TBS-brain extracts by category. Error bars represent mean. Statistical analyses used one-way-ANOVA with Dunn’s multiple comparisons test. *, *p* < 0.05. **G** Schematic of GM2A removing GM2 from the membrane for hydrolysis mediated by HEXA/HEXB. All 3 proteins were in the leading edge of the Diseases of metabolism term. **H** Scatter plot of GM2A across TBS-brain extracts by category reveal higher levels in AD. Error bars represent mean ﻿ ± SEM. Statistical analyses used one-way-ANOVA with Dunn’s multiple comparisons test. *, *p* < 0.05. **I** GM2A levels are elevated in CSF of AD compared to controls (data from [39]). Statistical analyses used unpaired t-test with Welch correction. *, *p* < 0.05

While associations of Aβ and tau were observed with effect size in the MEA and NI assays, neither could fully account for the magnitude of effects across brain samples in either assay. This is in accord with previous findings which showed that while Aβ and tau levels in human brain were elevated in those with AD, neither could fully account for the extent or rate of cognitive decline [43, 44]. Therefore, it is likely that additional factors in the human brain differentially impinge upon the toxicity of Aβ and tau either to accelerate degeneration or to protect against their effects. Therefore, we next used the same unbiased proteomic profiling data of these TBS-soluble brain extracts to identify proteins associated with neurotoxicity and neuroprotection in the MEA and NI assays. To assess the relationship between each of the 1841 proteins detected in this analysis and the in vitro phenotypes, Spearman’s correlation coefficients were calculated between the normalized LFQ value for each protein within each extract and the effect size of each extract in both the neurite integrity and spontaneous firing assays. Top correlations in each assay are shown in the waterfall plots (Fig. 6A, B). A positive correlation represents a protein that exhibits higher expression in extracts with less effect on neurite integrity or spontaneous firing, while a negative correlation represents a protein that exhibits higher expression in extracts with greater effect on these phenotypes. Shared effects were observed across both assays for 18 proteins (Fig. 6C).

Gene set enrichment analysis (GSEA) using the REACTOME database revealed that proteins associated with protection from toxicity shared terms that contained Rho GTPase effectors/activation of WASPs and WAVEs (Fig. 6D). Proteins driving protective associations include components of the WAVE regulatory complex (WRC). The WRC is a heteropentameric complex that interacts with the ARP2/3 complex to regulate actin polymerization. Interactions between the WRC and ARP2/3 complex mediate actin polymerization and branching, impacting neurite morphogenesis, plasticity, endocytosis, and trafficking. Leading edge proteins driving the GSEA enrichment include multiple proteins that are core components of both complexes (Fig. 6E, leading edge gene highlighted in red), and 4 of the top 18 shared associations between the NI and MEA assays were included under this category (ABI1, ABI2, BRK1, and ARPC1A). Intriguingly, TBS-soluble protein levels of both ABI1 and ABI2 were reduced in AD brain (Fig. 6F). ABI1 and ABI2 are not predicted to act extracellularly under physiological circumstances, but rather to be functioning inside cells. Thus, it is unclear here if the extracellular additions of these specific proteins present in brain extracts are *driving* a protective influence on neurons, or if the observed associations are simply biomarkers of the presence of other factors present in the brain that are mediating the effects. Future studies will further probe the meaning of these interesting associations.

Proteins associated with neurotoxic effects in both assays shared the enrichment term “Diseases of metabolism” (Fig. 6D). Intriguingly, many of the leading edge proteins driving this association have previously been implicated in neurodegenerative lysosomal glycosphingolipid storage disorders including GM2A, HEXA, HEXB, ASAH1, and GAA. Loss-of-function mutations in GM2A, HEXA and HEXB cause GM2 gangliosidosis, such as Tay-Sachs disease, B1 variant, Sandhoff disease, and GM2A deficiency (AB variant) (Fig. 6G, reviewed in [8, 45–47]. Of these proteins, the strongest association shared across the MEA and NI assays was with GM2A (Fig. 6C). GM2A can be found both in the cytoplasm and in the extracellular space, and its levels are elevated in TBS-soluble AD brain extracts (Fig. 6H) and in AD CSF (Fig. 6I, original CSF data from [48]), suggesting that extracellular GM2A may be physiologically relevant [8, 45–47].

### GM2A is sufficient to reduce mean firing rate and neurite integrity of human neurons

GM2A arose as a top candidate factor for exerting a causative influence on effect size in the MEA and NI assays, due to its association with neurotoxicity in both assays, its previously described role in neurodegenerative diseases, and its presence in the extracellular space in vivo. In order to test whether high levels of extracellular GM2A impart neurotoxic effects, we first treated human iNs with recombinant GM2A in the NI assay. Based on western blot quantification of GM2A in the human brain, we estimate that GM2A levels range from 0.5–1 ng/uL in this brain extract collection (Supplemental Fig. 2A). However, treatment with 2 or 5 ng/uL of recombinant GM2A had no effect in the NI assay (Supplemental Fig. 2B). GM2A has been shown to be post-translationally modified in multiple ways in mammalian cells [49], so we next tested human cell-derived GM2A in this assay. HEK293 cells were transfected with cDNA encoding human GM2A fused to GFP or else with a control plasmid. GM2A was endogenously expressed in HEK293 cells, but GM2A transfected cells expressed 2.5-fold higher levels over the endogenous levels (Supplemental Fig. 2C). On Day 21 of differentiation, human iNs were treated with GM2A-overexpressing (GM2A^OE^) or control TBS-lysates from these cells. GM2A^OE^ TBS lysates significantly reduced neurite integrity compared to control lysates (Supplemental Fig. 2D-G), suggesting that elevated extracellular GM2A can exert neurotoxic effects on human neurons.

**Fig. 7:**
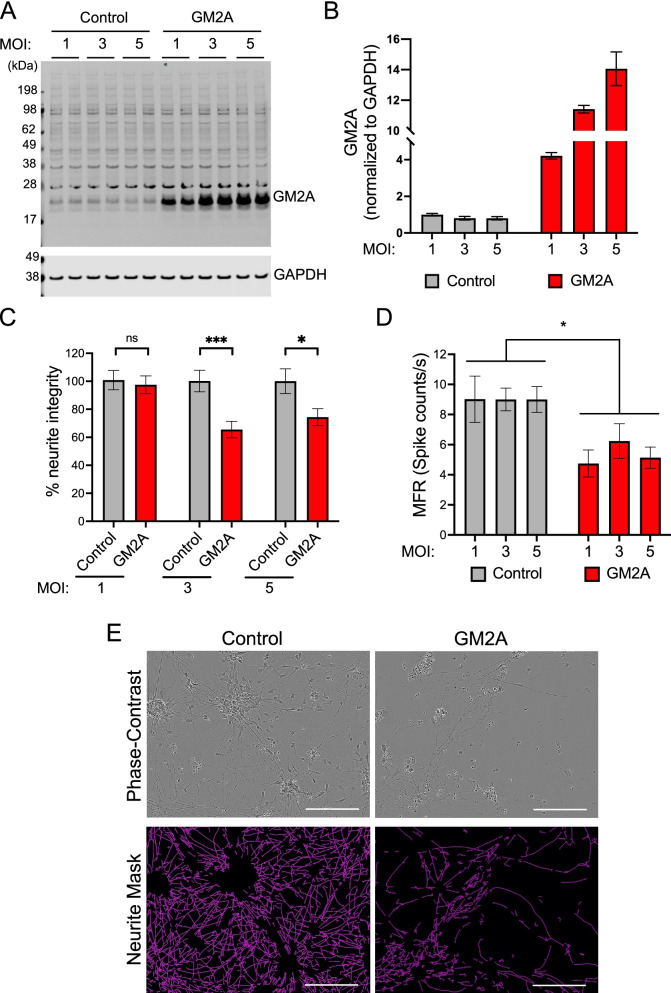
Elevated GM2A reduces neurite integrity and neuronal firing of human neurons. **A**, **B** Western blot of GM2A and GAPDH (A) and quantification of GM2A levels (B) in lysates from human iNs overexpressing GM2A or control at MOI = 1, 3, or 5. *N* = 2 for each condition. **C** Neurite integrity of Day 21 human iNs overexpressing GM2A or control at MOI = 1, 3, or 5. *N* = 90 for each condition. Error bars represent mean ﻿ ± SEM. Statistical analyses used unpaired t-test. *, *p* < 0.05; ***, *p* < 0.001; ns, not significant. **D** Spontaneous firing rate of Day 21 human iNs overexpressing GM2A or control at MOI = 1, 3, or 5. *N* = 8 for each condition. Error bars represent mean ﻿ ± SEM. Statistical analyses used paired t-test. *, *p* < 0.05. **E** Representative images of Day 21 human iNs overexpressing control or GM2A at MOI = 5. Scale bars = 200 μm

Having established that exogenously introduced GM2A could affect neurite integrity, we aimed to establish an experimental system to express GM2A within our human neuronal system. Lentivirus was employed to express untagged GM2A, and human neurons were transduced on Day 5 at increasing MOI (multiplicity of infection). Of note, this system is not directly comparable to extracellular addition of brain extracts containing elevated GM2A. Here, GM2A will be elevated by expression within the neurons themselves, resulting in an elevation of GM2A both in the lysosomes as well as in the extracellular space following release of lysosomes [8, 45–47]. Increasing protein levels of GM2A were expressed with increasing MOI, with 4- to 14-fold expression above endogenous levels (Fig. 7A, B). Human iNs were transduced with lentivirus encoding GM2A or else control lentivirus expressing GFP on Day 5 and analyzed on Day 21. GM2A overexpression resulted in a reduction in both neurite integrity and mean firing rate compared to control transduction, supporting the hypothesis that GM2A contributes to the neurotoxicity observed across our human brain lysate cohort (Fig. 7C-E). Additionally, although GM2A and AD brain extract (ALB01) did not reduce the overall protein expression level of presynaptic VGLUT2 (vesicular-glutamate transporter 2) or HOMER1 (homer protein homolog 1), a postsynaptic density scaffold protein (Supplemental Fig. 3A-G) in protein lysates, they did reduce the number of puncta with co-localization of VGLUT2 and HOMER1 in neurites (Supplemental Fig. 3H-L), indicating a potential impact of elevated GM2A on synaptic number in cultured neurons.

## Discussion

**Fig. 8:**
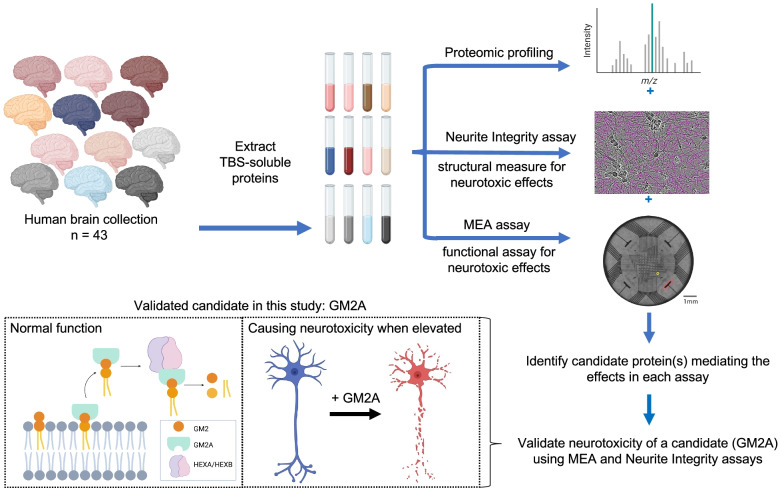
A system coupling proteomic profiling and functional assays identified GM2A as a neurotoxic factor to human brain

Proteomic studies of human brain tissue across large cohorts have identified a myriad of candidate factors that may be influencing AD risk and progression [16, 29, 30]. Next important steps are to test which of these factors are causal and which are indirect effects of disease processes. This study established and utilized two assays to assess neurotoxic and neuroprotective effects of soluble proteins present in human brain tissue (Fig. 8). Pathological findings in the human brain show progressive synapse loss along with the degeneration of neurites. Thus, we aimed to establish experimentally manipulable and well-controlled reductionist systems that quantitatively measure features of neurons that approximate processes relevant to electrophysiological activity (measuring activity using multi-electrode array) and to neurite integrity (using live cell imaging). We then used these assays to examine a cohort of aged human brains that span the clinical and pathological spectrum of aging relevant to AD. In this study, we chose to examine the TBS-soluble proteome of the postmortem brain in order to capture those factors that are present in the brain and available to act on neurites and synapses to induce neurodegeneration. However, these assays can be readily adapted to interrogate alternate fractions or subsets of proteins present in the brain.

One of the notable findings of this study is the heterogeneity of effects across brain samples, even within each category: low pathology, not cognitively impaired (LP-NCI), high pathology not cognitively impaired (HP-NCI), and AD. This heterogeneity was especially prominent in the MEA assay, with some brain samples in each category causing strong effects on activity while others had minimal effects. In the neurite integrity assay, while the LP-NCI samples had no effects, subsets of both HP-NCI and AD samples each induced varying levels of neurite degeneration. When data was aggregated within each category, HP-NCI and AD effect sizes were indistinguishable. These brain-specific differences in effects in these assays were mirrored by differences in the proteomic profiles, providing an opportunity to identify those proteins present in the brain samples that are contributing to neurotoxic and neuroprotective activities.

Using both primary rat cultured neurons (in the MEA assay) and human iNs (in the NI assay and the validation of neurotoxic GM2A by NI and MEA assays), we showed different types of neuronal cultures could be readily adapted to the systems. We began by using primary rat cortical neurons in the MEA assay for the human brain extract treatments since the electrophysiological characteristics of these cultures were well-defined through many decades of analyses (including optimization of the MEA system in our lab [7]). However, over the years of executing this study, extensive data were acquired regarding the human iN system, including data from our lab defining MEA characteristics of iNs over differentiation time [50]. Using an all-human system has several advantages for future studies that interrogate the impact of genetic background within the neurons assayed on responsiveness to brain extracts and specific candidate proteins.

These assays provide a platform to both identify candidate factors contributing to neurotoxicity and to test those factors in a reductionist system. In order to highlight candidate factors, we used association analyses to identify correlations between specific proteins and effect size. We first examined Aβ and tau, as numerous studies have previously shown that Aβ and certain forms of tau can have neurotoxic effects. Interestingly, overall Aβ levels did not associate with toxicity in either assay. Rather, the Aβ42:40 ratio associated with activity in the MEA assay while Aβ oligomer levels associated with neurite integrity. These findings are in line with previous studies showing that familial AD mutations consistently affect Aβ42:40 ratio, while effects on overall Aβ levels varies, and that oligomeric Aβ has neurotoxic effects while monomeric Aβ is not toxic [37, 40]. Extracellular tau has previously been reported to have neurotoxic effects [51]. Here, total tau levels were not associated with neurotoxicity while tau isoforms containing the N1/N2 inserts were associated.

The associations with Aβ and tau do not fully explain the effects observed in these assays in response to the panel of brain tissue extracts tested. Therefore, it was important to also take an unbiased approach to further probe the content of these brain extracts. Proteomic analyses of the set of brain extracts prioritized specific proteins differentially present in AD brain that also were associated either with neurotoxic activity or neuroprotection. In addition to the full-length intact proteins, we do not exclude the possibility that truncated or fragmented peptides in brain extracts also could be bioactive and cause neuronal toxicity. In addition, it is important to keep in mind that not all peptides and proteins present in these TBS-soluble brain extracts are normally present extracellularly. By virtue of the necessity of homogenization steps for the preparation of brain extracts, these extracts also will contain intracellular, cytosolic proteins that may also contribute to protective or neurotoxic effects when applied extracellularly.

As mentioned above, in addition to identifying candidates for neurotoxic effects, these assays also provide a platform to test hypotheses arising from these association studies. One of the top proteins associated in both assays was GM2A, and treatment of neurons with GM2A in the absence of brain extract induced a reduction in neurite integrity and mean firing rate, demonstrating an example of how these assays can be employed to test specific hypotheses arising from association analyses (Fig. 8). GM2A plays a key role in ganglioside turnover. It is essential for the degradation of GM2 by HEXA, and loss-of-function mutation results in a highly similar clinical presentation to Tay-Sachs disease. GM2A along with other sphingolipid activator proteins also aid in the degradation of other gangliosides and glycosphingolipids [8]. While deficiencies in GM2A cause fatal neurodegenerative disease, it was not previously appreciated that physiologically relevant elevations in levels of GM2A would directly affect the integrity of human neurons. Here we find that higher levels of GM2A are associated with neurotoxic effects in each assay, and there is an elevation in GM2A levels both in the AD brain and in CSF of AD patients. While GM2A is primarily active intracellularly modulating hexosaminidase activity in lysosomes, it also can be found extracellularly as a result of lysosomal fusion with the plasma membrane through lysosomal exocytosis [52]. However, the neutral pH of the extracellular space is predicted to substantially reduce the activity of these enzymes compared the acidic pH found in lysosomes. Future studies are warranted to determine the site(s) of action of GM2A in our experimental paradigms. Finally, gangliosides have previously been implicated in AD with GM1 at the plasma membrane affecting Aβ aggregation [53], and ganglioside-bound Aβ levels being associated with AD [54]. Interestingly, in the MEA assay, addition of both Aβ (42:40) and GM2A levels to a predictive model of effect size in that assay strongly improved the model (Supplemental Table 3), suggesting that these two factors may work in concert to induce neurotoxic effects.

## Conclusions

The techniques and results here introduce a systematic strategy to identify candidate proteins present in the aged human brain that potentially impact neuronal vulnerability. We validate GM2A as one factor that is elevated in the AD human brain that can exert neurotoxic effects on human neurons. The assays described herein and the data derived from these proof-of-principle experiments establish a platform for modeling neuronal vulnerability in response to factors endogenously present in the human brain and provide insights into proteins potentially contributing to AD pathogenesis.

## Supplementary Information

**Additional file 1: Supplemental Table 1.** Differentially expressed proteins in the soluble AD brain proteome. TBS-soluble extracts were prepared from brain tissue described in Table 1, and mass spectrometry used to quantify proteins. Differential expression of individual proteins was analyzed between AD vs. LP-NCI (A) and AD vs. HP-NCI (B). Many more proteins were differentially expressed in the LP-NCI vs. AD comparison than in the HP-NCI vs. AD comparison. Unpaired t-test with Welch correction, two-stage step-up, FDR < 0.05.

**Additional file 2: Supplemental Table 2.** Metrics obtained for each brain extract. **(A)** Quantifying Aβ, tau, and effect size in the neurite integrity and/or MEA assay for each brain extract. **(B)** Proteomic measurements of TBS-soluble brain tissue extracts tested in MEA and/or neurite integrity assay.

**Additional file 3: Supplemental Table 3.** Relevant results of linear modeling of effect size in neurite integrity and MEA assays.

**Additional file 4: Supplemental Fig. 1.** Effects of human brain extracts on spontaneous firing of cultured cortical neurons are heat labile and are strongest in a high molecular weight fraction. (**A**) Images of primary rat cortical cultures stained for neuronal markers TAU and DAPI the nuclear marker following 4-day treatment with control brain extract (MGH1887) or AD brain extract (ALB01). Scale bars = 100 μm. (**B**) Titration of AD brain extract (ALB01, red) compared to an LP-NCI brain extract (MGH1887, blue) at 4-days post-treatment in primary rat cortical neurons. (**C**) Denaturing extracts by boiling rescues effects on spontaneous firing Spontaneous firing activity within rat cortical cultures following 4-day treatment with native (open points) or boiled (shaded points) human brain extracts. Each point represents a single multi-electrode array, bars represent mean ﻿ ± SEM. Significant differences between conditions indicated by brackets and associated *p*-value. **Supplemental Fig. 2.** Generation and testing of cell-derived GM2A in neurite integrity assay. (**A**) Western blot probed for GM2A measuring GM2A levels in TBS-soluble brain extracts from 4 individuals: (1) MGH1887, (2) MGH1837, (3) RM304, and (4) RM305. Serial diluted recombinant GM2A ranging from 0.005 to 0.05 μg served as standards. GM2A is estimated to be ~ 1 ng/uL in the brain extracts. (**B**) Neurite integrity of Day 21 human iNs treated with 2 or 5 ng/uL recombinant GM2A was comparable to that of untreated iNs (0 ng/uL recombinant GM2A). (**C**) Western blot probed for GM2A measuring GM2A levels in TBS-soluble protein lysates from HEK293 transfected with GM2A or control cDNA construct. Lysates from 2 independent transfections of the GM2A construct were measured. Serial diluted recombinant GM2A ranging from 0.0025 to 0.01 μg served as standards. Total GM2A (endogenous and GFP-tagged) is estimated to be ~ 25 ng/uL in the TBS-soluble protein lysates from GM2A-transfected HEK293 cells. (**D-E**) Neurite integrity of Day 21 human iNs treated with 0.25 or 0.5 ng/uL HEK293-derived, TBS-soluble GM2A started to show significant reduction 4 hr. after the treatment (E) and GM2A-mediated reduction of neurite integrity is dose-dependent (D&E). *N* = 10 for each condition. Statistical analyses used unpaired t-test at each time point with Holm-Šídák method. *, *p* < 0.05; **, *p* < 0.01; ****, *p* < 0.0001. (**F-G**) Neurite integrity of Day 21 human iNs treated with TBS-soluble protein lysates derived from HEK293 cells transfected with GM2A or control cDNA construct. Neurite integrity assessed every 4 hrs (F) and 24-hr post-treatment (G) are presented. *N* = 15 for each condition. Error bars represent mean ﻿ ± SEM. Statistical analyses used unpaired t-test at each time point with Holm-Šídák method. **, *p* < 0.01. **Supplemental Fig. 3.** Expression of synaptic proteins in neurons with elevated GM2A. **(A-G)** Western blot probed for VGLUT2, HOMER1, GM2A, and GAPDH in Day 21 human iNs overexpressing GM2A or control at MOI = 5 (transduced on Day 5), and in Day 21 human iNs treated with AD brain extract (ALB) or control (treated from Day 17). *N* = 5 for each condition. **(H-L)** Images of VGLUT2 and HOMER1 immunostains in Day 21 human iNs overexpressing GM2A or control at MOI = 5 (transduced on Day 5), and in Day 21 human iNs treated with AD brain extract (ALB) or control (treated from Day 17). The insets show the quantified, representative neuritic segments (*N* = 15 for each condition). Scale bars: 20 μm. Statistical analyses used unpaired t-test. *, *p* < 0.05; **, *p* < 0.01; ****, *p* < 0.0001; ns, not significant.

## Data Availability

The datasets generated during the current study are available in the Supplementary Materials. The results published here are in part based on human brain tissue data obtained from the AMP-AD Knowledge Portal (https://adknowledgeportal.synapse.org/). Study data were provided by the Rush Alzheimer’s Disease Center, Rush University Medical Center, Chicago. ROS and MAP data can be requested at https://www.radc.rush.edu.

## Abbreviations

Aβ: amyloid beta
AD: Alzheimer’s disease
ANCOVA: analysis of covariance
ANOVA: analysis of variance
BCA: bicinchoninic acid
CSF: cerebrospinal fluid
DEP: differentially expressed protein
DMEM: Dulbecco’s Modified Eagle’s Media
ELISA: enzyme-linked immunosorbent assay
FAD: familial Alzheimer’s disease
FDR: false discovery rate
GM2A: ganglioside GM2 activator
GSEA: gene set enrichment analysis
GuHCl: guanidine hydrochloride
HP-NCI: high pathology and non-cognitively impaired
iNs: induced pluripotent stem cell-derived neurons
LFQ: label-free quantification
LME: linear mixed effect
LC/MS-MS: liquid chromatography/tandem mass-spectroscopy
LOF: loss-of-function
LP-NCI: low pathology and non-cognitively impaired
MOI: multiplicity of infection
MTBR: microtubule-binding repeat
MEA: multi-electrode array
MFR: mean firing rate
NFT: neurofibrillary tangles
NI: neurite integrity
PMI: postmortem interval
PCA: principal components analysis
RMS: root mean squared
SD: standard deviation
TBS: tris buffered saline
VSN: variance stabilization normalization
WRC: wave regulatory complex
