# Supplementary figures and images for: Elevated ganglioside GM2 activator (GM2A) in human brain tissue reduces neurite integrity and spontaneous neuronal activity
Source: Mol Neurodegener. 2022 Sep 21;17:61. doi: 10.1186/s13024-022-00558-4 (PMC9494921; doi:10.1186/s13024-022-00558-4)

Supplemental Figure 1.

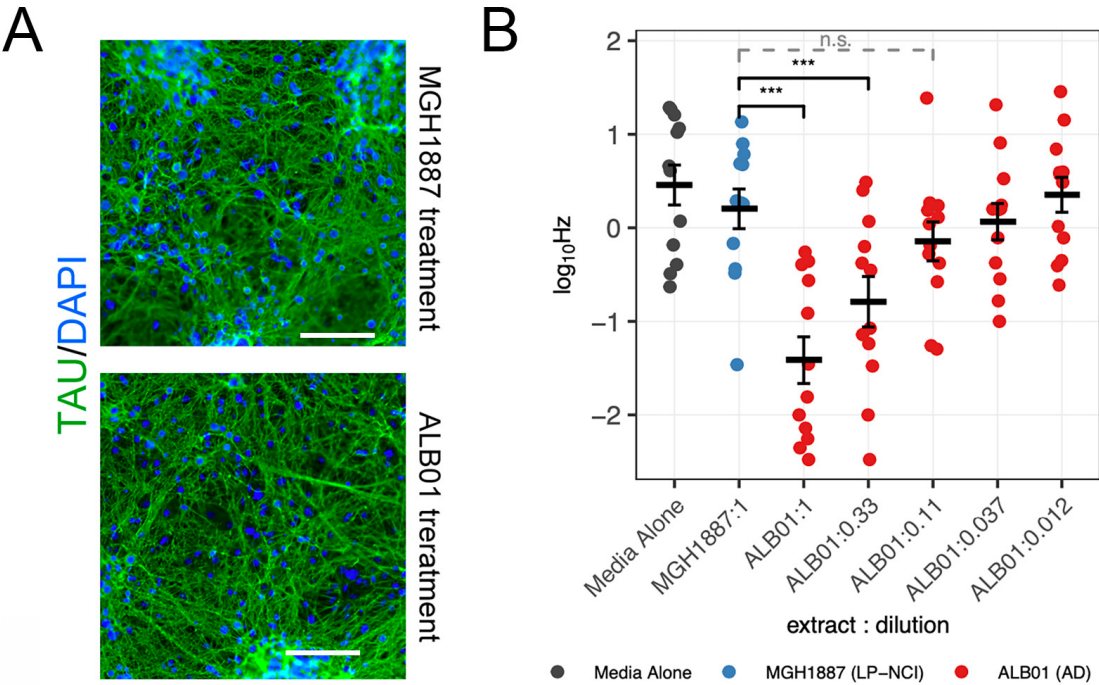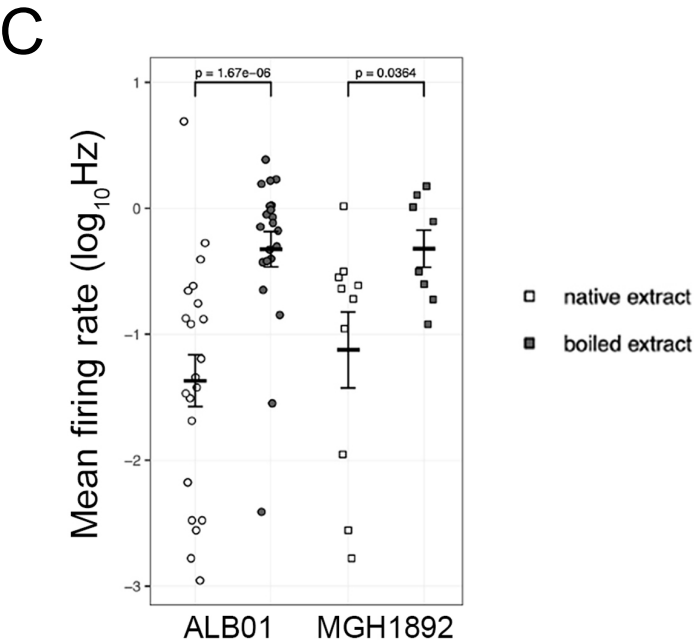

Supplemental Figure 2.

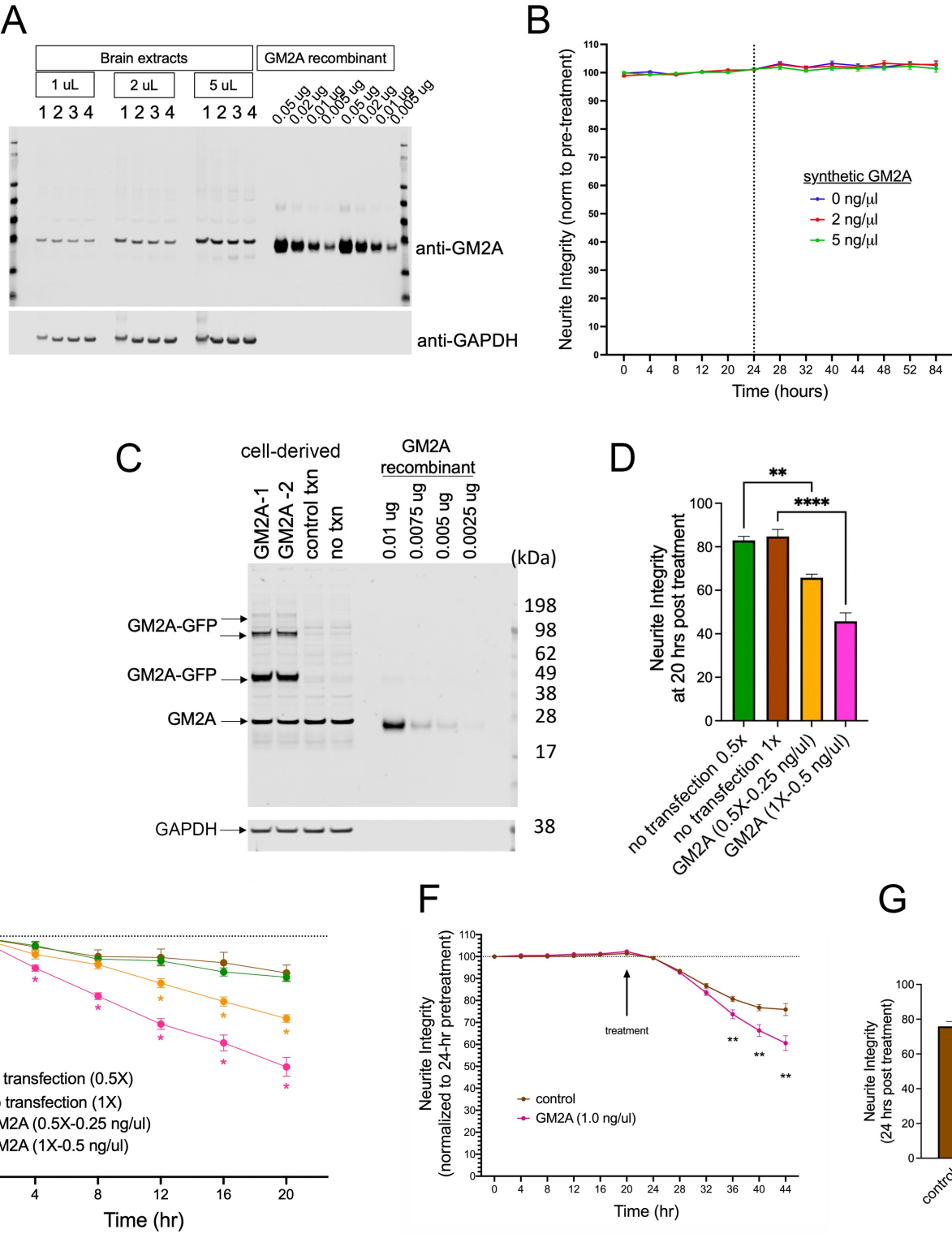

Supplemental Figure 3.

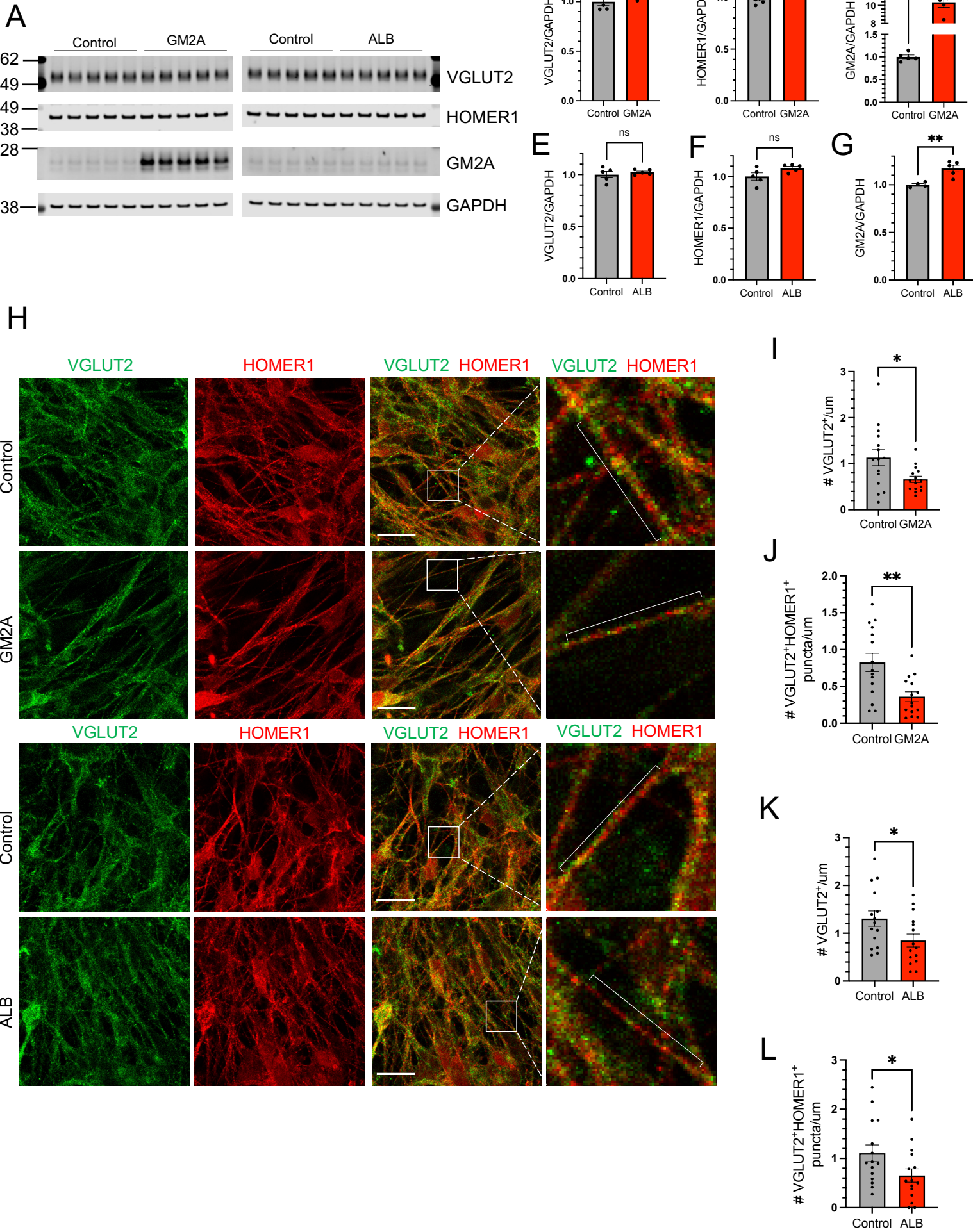

Supplement: Supplementary file 4 — Additional file 4: Supplemental Fig. 1. Effects of human brain extracts on spontaneous firing of cultured cortical neurons are heat labile and are strongest in a high molecular weight fraction. (A) Images of primary rat cortical cultures stained for neuronal markers TAU and DAPI the nuclear marker following 4-day treatment with control brain extract (MGH1887) or AD brain extract (ALB01). Scale bars = 100 μm. (B) Titration of AD brain extract (ALB01, red) compared to an LP-NCI brain extract (MGH1887, blue) at 4-days post-treatment in primary rat cortical neurons. (C) Denaturing extracts by boiling rescues effects on spontaneous firing Spontaneous firing activity within rat cortical cultures following 4-day treatment with native (open points) or boiled (shaded points) human brain extracts. Each point represents a single multi-electrode array, bars represent mean ﻿ ± SEM. Significant differences between conditions indicated by brackets and associated p-value. Supplemental Fig. 2. Generation and testing of cell-derived GM2A in neurite integrity assay. (A) Western blot probed for GM2A measuring GM2A levels in TBS-soluble brain extracts from 4 individuals: (1) MGH1887, (2) MGH1837, (3) RM304, and (4) RM305. Serial diluted recombinant GM2A ranging from 0.005 to 0.05 μg served as standards. GM2A is estimated to be ~ 1 ng/uL in the brain extracts. (B) Neurite integrity of Day 21 human iNs treated with 2 or 5 ng/uL recombinant GM2A was comparable to that of untreated iNs (0 ng/uL recombinant GM2A). (C) Western blot probed for GM2A measuring GM2A levels in TBS-soluble protein lysates from HEK293 transfected with GM2A or control cDNA construct. Lysates from 2 independent transfections of the GM2A construct were measured. Serial diluted recombinant GM2A ranging from 0.0025 to 0.01 μg served as standards. Total GM2A (endogenous and GFP-tagged) is estimated to be ~ 25 ng/uL in the TBS-soluble protein lysates from GM2A-transfected HEK293 cells. (D-E) Neurite integrity of Day 21 hum [file 13024_2022_558_MOESM4_ESM.pdf]
